# Expression of Concern on ‘Hypoxic stress suppresses RNA polymerase III recruitment and tRNA gene transcription in cardiomyocytes’

**DOI:** 10.1093/nar/gkag936

**Published:** 2026-09-28

**Authors:** Julian E Sale, Barry L Stoddard

This is an Expression of Concern regarding: Isabelle Ernens, Sarah J. Goodfellow, Fiona Innes, Niall S. Kenneth, Louise E. Derblay, Robert J. White, Pamela H. Scott, Hypoxic stress suppresses RNA polymerase III recruitment and tRNA gene transcription in cardiomyocytes, Nucleic Acids Research, Volume 34, Issue 1, 1 January 2006, Pages 286–294, https://doi.org/10.1093/nar/gkj402

In August 2026, the Editors were made aware of concerns regarding potential image irregularities in Figure 4A of the above article, which were also raised on PubPeer (https://pubpeer.com/publications/774465B238E111AE3636123033ADC0). These concerns relate to the apparent duplication of immunoblot bands depicting different experimental conditions or proteins.

The Editors have contacted the authors and have escalated to the institution for investigation in line with COPE guidance. In the interim, the Editors are publishing this Expression of Concern to alert readers while the outcome of the investigation is pending and advise readers to examine the details of this study with particular care.

